# A Comprehensive Metabolomics Analysis of Fecal Samples from Advanced Adenoma and Colorectal Cancer Patients

**DOI:** 10.3390/metabo12060550

**Published:** 2022-06-15

**Authors:** Oiana Telleria, Oihane E. Alboniga, Marc Clos-Garcia, Beatriz Nafría-Jimenez, Joaquin Cubiella, Luis Bujanda, Juan Manuel Falcón-Pérez

**Affiliations:** 1 Exosomes Laboratory, CIC bioGUNE-BRTA, CIBERehd, Bizkaia Technology Park, 48160 Bilbao, Spain; 2 Metabolomics Platform, CIC bioGUNE-BRTA, CIBERehd, Bizkaia Technology Park, 48160 Bilbao, Spain; oalboniga@cicbiogune.es; 3 LEITAT Technological Center, C/de la Innovació, 2, 08225 Terrassa, Spain; marc.clos.garcia@sund.ku.dk; 4 Osakidetza Basque Health Service, Donostialdea Integrated Health Organisation, Clinical Biochemistry Department, 20014 San Sebastian, Spain; beatriz.nafriajimenez@osakidetza.eus; 5 Department of Gastroenterology, Complexo Hospitalario Universitario de Ourense, Instituto de Investigación Sanitaria Galicia Sur, Centro de Investigación Biomédica en Red de Enfermedades Hepáticas y Digestivas (CIBERehd), 32005 Ourense, Spain; joaquin.cubiella.fernandez@sergas.es; 6 Department of Gastroenterology, Hospital Donostia/Instituto Biodonostia, Centro de Investigación Biomédica en Red de Enfermedades Hepáticas y Digestivas (CIBERehd), Universidad del País Vasco (UPV/EHU), 20014 San Sebastián, Spain; luis.bujandafernandezdepierola@osakidetza.eus; 7 IKERBASQUE, Basque Foundation for Science, 48011 Bilbao, Spain

**Keywords:** untargeted metabolomics, colorectal cancer, faecal samples, biomarkers

## Abstract

Accurate diagnosis of colorectal cancer (CRC) still relies on invasive colonoscopy. Noninvasive methods are less sensitive in detecting the disease, particularly in the early stage. In the current work, a metabolomics analysis of fecal samples was carried out by ultra-high-performance liquid chromatography–tandem mass spectroscopy (UPLC-MS/MS). A total of 1380 metabolites were analyzed in a cohort of 120 fecal samples from patients with normal colonoscopy, advanced adenoma (AA) and CRC. Multivariate analysis revealed that metabolic profiles of CRC and AA patients were similar and could be clearly separated from control individuals. Among the 25 significant metabolites, sphingomyelins (SM), lactosylceramides (LacCer), secondary bile acids, polypeptides, formiminoglutamate, heme and cytidine-containing pyrimidines were found to be dysregulated in CRC patients. Supervised random forest (RF) and logistic regression algorithms were employed to build a CRC accurate predicted model consisting of the combination of hemoglobin (Hgb) and bilirubin E,E, lactosyl-N-palmitoyl-sphingosine, glycocholenate sulfate and STLVT with an accuracy, sensitivity and specificity of 91.67% (95% Confidence Interval (CI) 0.7753–0.9825), 0.7 and 1, respectively.

## 1. Introduction

Colorectal cancer (CRC) is the third-most common malignant neoplasm worldwide in men and the second one in women, representing almost 10% of global cancer incidence, and is the second leading cause of cancer-related death [1]. In Spain, colorectal neoplasm is the second leading cancer among men (after lung cancer) and women (after breast cancer), accounting for a total of 12,010 (10.6%) deaths [1]. Screening and early detection are excellent measures to prevent colorectal cancer and associated death [2]. Following the 2003 European Guidelines and the National Strategy against Cancer of 2006, CRC screening is based on the detection of occult blood in feces (FOB) using noninvasive guaiac-based test (gFOBT) and biennial quantitative fecal immunochemical test (FIT), and an invasive colonoscopy under sedation for FIT positive cases [3]. However, due to unsatisfactory sensitivity and specificity, FOB has still limited the clinical application in CRC diagnosis [4,5] and an invasive standardized traditional optical colonoscopy is still the gold-standard method to diagnose CRC, which has several risks attributed to the intervention [6]. For these reasons, high-throughput ‘omics’ technologies such as metabolomics can be considered as an important tool for biomarker discovery for disease diagnosis and prognosis [7,8,9,10,11]. Metabolomics, which focuses on the detection and quantitation of small molecules (metabolites) in different specimens [12,13,14], is the omic technology that best mirrors the human phenotype, as metabolites are present in all biological structures and metabolic pathways [15,16,17,18,19,20]. Although metabolomics is a technique that has reached popularity in the last few decades and has been applied in a wide range of diseases including cancer, it is still in the pioneering phase in CRC research [12]. Even though the improvement of the knowledge on dysregulated biomarkers associated to cancer is necessary, metabolomics has pointed out several identified biomarkers for CRC and cancer diagnosis that, at the same time, can be used for therapeutic evaluation [13,18,21,22,23].

In the current study, the main objective is to study and compare the fecal metabolomic profile of patients with normal colonoscopy, advanced adenoma (AA) and CRC to obtain candidate biomarkers and predictive models capable to identify the different disease stages.

## 2. Results

After quality assurance and data processing, a total of 1380 metabolites were detected in 120 fecal samples, derived from benign (*n* = 40, control), clinically localized colorectal cancer (*n* = 40, CRC) and advanced adenoma (*n* = 40, AA), that fulfill the Metabolon’s acceptance criteria, being 6% of RSD for instrument variability and 10% of RSD for total process variability, in the four methods described in the Section 4.

Using the Metabolon’s in-house library and library entries of purified standards, 229 amino acids, 34 carbohydrates, 55 cofactor and vitamins, 12 metabolites of energy production metabolism, 353 lipids, 63 nucleotides, 12 partially characterized nucleotides, 43 peptides and 241 xenobiotics metabolites make a total of 1042 compounds with known structural identity and 338 with unknown structures (Supplementary Table S1).

### 2.1. Univariate, Multivariate and Logistic Regression Analysis

**Figure 1:**
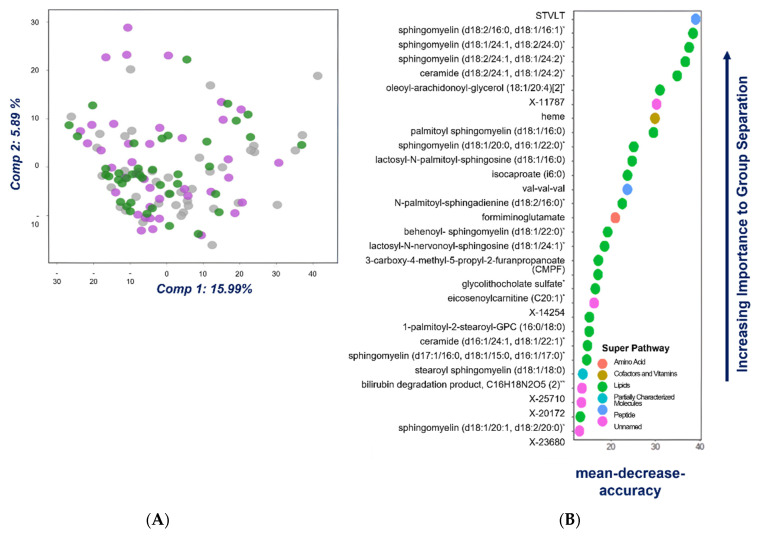
Two-dimensional principal component analysis plot for all fecal samples color-coded by group (grey—control group; purple—AA; and green—CRC) (**A**). Top 30 metabolites’ biochemical importance plot performed by RF classification-method analysis for control + AA vs. CRC. The plot shows each variable on the Y-axis and their importance on the X-axis (**B**). * indicates the compound has not been confirmed based on standard, but highly confident on its identification, and ** standard was not available and reasonably confident on its identification.

The 1380 metabolites were used for the analysis by principal component analysis (PCA). As it can be seen in PCA scores plot (Figure 1A) no tendency or separation was observed among the three groups. Considering this fact, a supervised analysis was performed by the random forest (RF) method. Firstly, the three groups were compared but the predictive accuracy obtained in the random forest confusion matrix was 52% (Figure S3 in the Supplementary Material). Nearly 50% of normal individuals were assigned as AA and vice versa, and 60% of CRC patients were correctly assigned. 

Considering the RF’s poor ability when it comes to differentiating the three groups, a new RF was performed, fusing on the one hand AA + CRC compared with the control group, and on the other hand grouping control + AA compared with CRC. In both cases the predictive accuracy increased, reaching 75% in the last case, suggesting that the metabolic profiles of AA samples might be similar to controls (Supplementary Material Figure S4). Considering these results, the important variable for control + AA vs. CRC was estimated using RF, where Figure 1B gathers the top 30 metabolites’ biochemical importance plot, as well as the class of each compound. As can be seen, most of the compounds belong to the lipid class, indicating that mainly lipid metabolism is dysregulated in CRC patients.

Finally, a univariate statistical test based on Welch’s two-sample *t*-test was performed comparing CRC with control and AA individuals. In total, 25 metabolites had *q*-values ≤ 0.05 and were identified, except one (Table 1). Among them, 17 were upregulated and 8 were downregulated for CRC patients compared to control and AA individuals. In Table 1, the significant values obtained after FDR application in the Welch’s two-sample *t*-test are summarized. The identified compounds and their associated pathway, as well as the fold change and the identification confidence level based on the metabolomics society initiative (MSI), are included. Comparing univariate and multivariate statistical results of control + AA vs. CRC, 20 out of 25 significant metabolites obtained by Welch’s *t*-test were also found to be important variables by RF classification method. This makes the results reliable, as different statistical approaches reached same significant metabolites.

A logistic regression was also built, including patient demographic data in the model. The combination of hemoglobin (Hgb) concentration in fecal samples with four metabolites predicts CRC with predictive accuracy of 91.67% (95% Confidence Interval (CI) 0.7753–0.9825), 0.7 of sensitivity and 1 of specificity. The prediction model was:

Y = −4.0684 + 0.7050 Hgb − 0.2694 Bilirubin E,E + 0.7248 lactosyl-N-palmitoyl-sphingosine (d18:1/16:0) − 0.3435 glycocholenate sulfate + 0.4484 STLVT.

Hgb concentration in fecal samples, lactosyl-N-palmitoyl-sphingosine (d18:1/16:0) and STLVT were positively associated with risk of developing CRC. In contrast, inverse associations with risk of developing CRC were observed to Bilirubin E,E and glycocholenate sulfate. 

**Figure 2:**
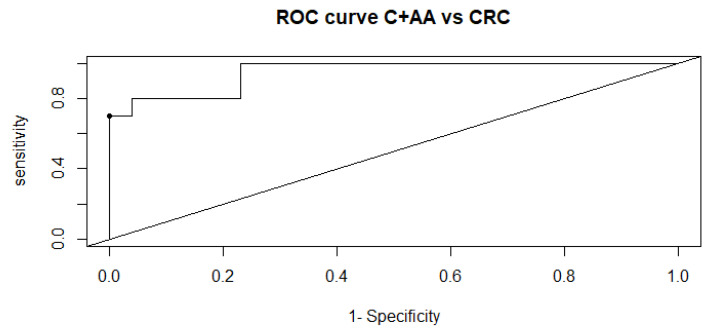
ROC curve of predictive model using logistic regression with Hgb and predicted metabolites.

The obtained AUC value was 0.9500 (95% CI 0.8802-1) in the predicted ROC curve, and the best threshold was achieved at 0.678 (Figure 2).

### 2.2. Comparison of Metabolome of Colorectal Cancer, Advanced Adenoma and Control Groups 

As can be observed from Table 1, lipid metabolism was the most affected pathway, given that it stands for 60% of the altered metabolites, specifically metabolites involved in the sphingolipid (SL) pathway. Ceramides (Cer) are the central molecules in SL metabolism, which are produced by both catabolic and anabolic mechanisms, thereby crafting a metabolic hub, obtaining sphingomyelins (SM) via SMase pathway, glycosphingolipids (GSL), hexosylceramide (HexCer) and lactosylceramides (LacCer) via cerebroside pathway and sphingosine via salvage pathway [24]. Lactosylceramides were significantly found not only by Welch’s two-sample *t*-test, but also lactosyl-N-palmitoyl-sphingosine (d18:1/16:0) was found as part of the prediction model for CRC disease by logistic regression. 

According to our results, SM reflected a generalized increase in CRC samples compared to those from control and AA. Remarkably SM 34:1 and SM 42:3 molecules, since both are related with Cer 34:1 and Cer 42:3 significantly increased not only in control + AA vs. CRC even in CRC comparing with AA too (Table 1). The same results were obtained for LacCer 34:1 and LacCer 42:3, but the HexCer (intermediate molecules between Cer and LacCer) do not show a statistical significance, but a slight tendency to increase was observed. Another interesting finding that is consistent with these results and findings, even though statistical significance was not achieved, is that 3-ketosphinganine (Supplementary Table S1), which is one of the Cer precursors obtained by De Novo pathway, was also increased in control samples in comparison with both AA or CRC groups. 

Apart from lipids, the amount of hemoglobin-derived heme group measured in stool samples from CRC patients was significantly higher compared to those amounts obtained from AA and control individuals, even in the control + AA fusion group. Other heme-related compounds (e.g., bilirubin) or hemoglobin fragments of hydrolyzed α-chain STVLT (α133–137) and VGAHAGEY (α17–24) [25], the bilirubin E,E, were reduced significantly, and STVLT increased in CRC individuals. For the VGAHAGEY metabolite a very high fold change was observed in CRC samples compared with another groups; even so, the change was not statistically significant (Supplementary Table S1). 

We could observe in CRC samples a reduction tendency in the levels of secondary-bile-acid metabolites, but only two metabolites associated with secondary-bile-acid metabolism were statistically significant in CRC individuals compared to AA and control + AA. Apart from these metabolites, it was also observed that other metabolites involved in primary-bile-acid or secondary-bile-acid metabolism were altered in CRC patients (*q*-value ≥ 0.05). 

A downregulated tendency was observed in pyrimidine-related metabolites (e.g., cytidine, cytosine, 3-ureidopropionate, 3-ureidoisobutyrate and 3-aminoisobutyrate) especially in cytidine, since that was the unique nucleotide that was statistically significant in CRC samples compared with the control and control + AA group. 

Finally, considering amino-acid metabolism, formiminoglutamate (FIGlu) was found to be significant, with an upregulated tendency in CRC patients compared to AA and control + AA. This metabolite is an important intermediate metabolite that was finally involved in biological pathways such as tricarboxylic acid (TCA) cycle, and by its conversion in several steps into alpha-ketoglutaric acid also found upregulated in CRC patients (Supplementary Table S1), and purine synthesis.

## 3. Discussion

Nowadays, colonoscopy is the most reliable standard for CRC detection, despite being an invasive method. For this reason, we proposed metabolomics as an approach for CRC early detection as it is emerging as an efficient approach for the detection of different tumors [26,27,28,29]. 

Our previous metabolomics approach studied the differences in 105 fecal metabolites, including glycerolipids, glycerophospholipids, sterol lipids and sphingolipids. Although 18 metabolites were significantly altered in patients with advanced neoplasia compared to controls, the combination of cholesteryl esters (ChoE (18:1), ChoE (18:2) and ChoE (20:4)), phosphatidyl ethanolamines (PE (16:0/18:1)), sphingomyelins (SM (d18:1/23:0) and SM (42:3)) and triglyceride (TG (54:1)) discriminated AA and CRC from control patients [7]. Now, applying a multiplatform untargeted metabolomics with four metabolite-extraction processes and analyzed by four different UPLC-MS/MS methods, we could extend our previous study to 1380 metabolites, increasing the coverage and obtaining a wider picture of metabolic dysregulation. 

Most of the recent epidemiological studies focused on the role of dietary heme in the pathogenesis of CRC [30,31,32,33]. Heme induced DNA damage and proliferation of human colonic epithelial Caco-2 cells via H_2_O_2_ produced by heme oxygenase (HO), suggesting that HO-1 and cell proliferation or apoptosis are linked [30,31,32,33,34], where high expression of HO-1 has been observed in solid tumors in humans [30,35,36]. In our study, we detected higher levels of the heme group in CRC samples, further supporting this association of heme and CRC. 

In addition of its primordial function as an oxygen carrier, hemoglobin is also a source of endogenous bioactive heme-peptides [37] STVLT and VGAHAGEY, related to antimicrobial peptides (AMPs) that are indispensable components of the innate immune system in various species, including humans, animals and plants, and become the first-line defense against foreign attacks [37,38,39]. AMPs have a broad spectrum of biological activities, including antibacterial, antifungal, antivirus and anticancer [37,40,41,42]. The presence of STVLT or VGAHAGEY, and especially both, indicates a high probability of being CRC [43]; and the absence of both indicates a high likelihood not to be CRC [43,44]. In agreement with this, we detected higher levels of those heme-related peptides in the CRC samples.

This study found that 60% of the detected significant metabolites that were upregulated belonged to metabolites from lipid-metabolism pathways, highlighting the critical role of this metabolic route in tumoral biology, since changes in lipid metabolism can affect numerous cellular processes [45].

The dysregulated lipid metabolism, and in particular SL metabolism, is a consequence of the cell growth, mortality and invasion currently occurring in tumoral environments that could act as tumor biomarkers [46,47]. Cancer cells can further support their proliferation, metastasis and resistance to chemotherapeutics by upregulating the production of pro-survival SLs, such as sphingosine-1-phosphate, and downregulating pro-cell death SLs such as Cer [24]. 

Cer can be generated by several mechanisms, including catabolic and anabolic pathways [24,45,46,47,48,49]. The results showed that CRC patients have decreased levels in 3-ketosphinganine, which is an endogenous Cer precursor synthesized through “de novo” pathways. The decrease in this metabolite is associated with cell proliferation [50,51,52,53,54,55].

Cer are precursors of some SL, which GCS can glycosylate to obtain glucolipids as HexCer and a posteriori LacCer or phospholipids as SM by SMases. The modulations of HexCer and SM levels are associated with cell life/death, and the accumulation of LacCer is associated with cell proliferation [50,56,57,58]. LacCer are key metabolites in several biological functions such as immunological response. It is believed that proinflammatory factors activate LacCer synthase to generate LacCer, which activates “oxygen-sensitive” signaling pathways affecting such cellular processes as proliferation, migration, adhesion, etc. Dysregulation in these pathways can affect several diseases of the cardiovascular system, cancer and inflammatory states. Thus, LacCer metabolism is a potential target for new therapeutic treatments and a more targeted approach for future studies [59].

SM, part of phospholipids, is an integral part of the membrane and determines its structure. SM comprises the most significant proportion of SL, among which the d18 base backbone is the dominant species. Several studies have demonstrated that the increase in phospholipids in the cell membrane are related to carcinoma [58]. It has been suggested that higher levels of phospholipids can be due to enhanced cell-membrane synthesis related to accelerated neoplasm cell replication [58,60]. In the first phase, G1, of the cell cycle, are observed the most remarkable changes in phosphatidylcholine and phosphatidylethanolamine content, where biosynthesis, catabolism and metabolism of phospholipids are controlled by enzyme activity at its maximum level [58,61,62], and differences in membrane phospholipid contents can influence metastasis development [58,63]. 

Another parameter to consider is the connection between cancer and lipid metabolism with the diet effect. High-fat diets can promote the hepatic synthesis of cholesterol-derived bile acids (BAs) and increase their delivery to the colonic lumen [8,64,65,66,67]. After secretion to the intestinal lumen, primary bile acids are deconjugated, and most of them are reabsorbed in small-intestinal transit. The remaining BAs enter the colon. High-fat diets stimulate the growth and activity of bacteria with 7α-dehydroxylation capacity, converting primary bile acids into secondary bile acids associated with tumorigenic activity [8,56]. High concentrations of secondary bile acids in the feces, blood and bile have been linked to the pathogenesis of colon cancer [8,26,53,68,69,70]. Here, we observed a disturbance of BAs metabolism, and in contrast to previous studies that suggested that BAs cause DNA damage and are promoters of colon carcinogenesis [8,26,53,71,72], our research showed that compared with AA and control samples, BAs decreased in CRC individuals above all in glycolithocholate sulfate and glycocholenate sulfate. Considering that only two metabolites were significant, and the importance of this pathways in cancer disease, this finding here opens a more targeted approach on bile-acid metabolism to enhance the biological snapshot associated to colorectal cancer.

Some studies describe the direct association between gut microbiota and metabolome, finding differences in fecal-bacterial compositions between patients with and without adenoma [26,53,64]. In particular, bile-acid metabolism showed significant correlations with genera from the *Firmicutes* phylum (Clostridium, Dehalobacterium, Ruminococcus and Oscillospira) and a genus from the *Actinobacteria* phylum (Adlercreutzia), and sphingolipid metabolism showed negative correlations with Dehalobacterium, Ruminococcus and Oscillospira [53]. Although is known that diet (e.g., fatty-acid content), host physiology (body mass index) and immune response are indirectly connected, we did not study this association due to general Spanish population was considered for this study.

Finally, the finding observed for the formiminoglutamate (FIGlu) is remarkable. The increased tendency in CRC patients was related to alterations in tricarboxylic acid (TCA) cycle, key pathways for energy production and the synthesis of purine, pyrimidine, amino acids, etc. intermediates, as well as purine synthesis. Briefly, FIGlu is an intermediate metabolite of the pathway that converts histidine into glutamic acid and depends on tetrahydrofolate (THF), a key compound in one-carbon metabolism. FIGlu is converted into glutamic acid and into 5,10-methenyl-THF by the action of the formiminotransferase. On one hand, glutamic acid enters in the tricarboxylic acid (TCA) cycle as alpha-ketoglutaric acid. On the other hand, 5,10-methenyl-THF is a metabolite directly used for purine synthesis as it acts as a carbon donor. Both pathways, TCA cycle and purine synthesis, were found to be altered in certain cancer cells [73,74] and could explain the enhancement on cellular proliferation. 

The RF method showed the best results for C + AA vs. CRC, and hence the generalized regression model was performed evaluating this model, analyzing accuracy, sensitivity, specificity, AUC, etc. The model predicted a combination of Hgb along with Bilirubin E,E, lactosyl-N-palmitoyl-sphingosine (d18:1/16:0), glycocholenate sulfate and STLVT as CRC predictor.

In summary, although the number of samples is limited, our comprehensive metabolomics study showed alterations in several metabolisms involving lipids, cofactors, polypeptides and nucleotides in CRC patients. Hgb-related molecules, four metabolites (Bilirubin E,E, lactosyl-N-palmitoyl-sphingosine (d18:1/16:0), glycocholenate sulfate and STLVT), microbiome and BAs metabolism are potentially valuable for future research in the diagnosis and prevention of colorectal cancer in an extensive cohort-study validation incorporated in clinical trials as potential biomarker. Given that this study was performed with samples collected from regular population screenings, some limitations about the collection of the information related to diet, lifestyle and diurnal variations were not included in the study protocol, and the influence of those parameters will need to be addressed in future investigations.

## 4. Materials and Methods

### 4.1. Clinical Samples and Study Population

Samples were obtained for the “metabolomic profile for the diagnosis of colorectal cancer and its precursor lesion, advanced adenoma” study, from patients submitted to colonoscopy. They donated the samples to the biobank of Instituto de Investigación Sanitaria Galicia Sur. The study was approved by Drug Research Ethical Committee (CEIm-G) (Code 2019/411). Patients self-collected a fecal sample from one bowel movement without specific diet or medication restrictions the week before the colonoscopy [75]. The fecal sample was brought to the laboratory in less than 4 h, split in aliquots and immediately frozen at −80 °C. One aliquot was shipped to Metabolon, Inc (Metabolon, Inc., Durham, NC, USA) for analysis and other aliquot was employed for FOB measurement using SENTIFIT^®^ FOB Gold Latex fecal immunoassay test (FIT) (Sentinel Diagnostics, Castellana G. BA, Italy). A total of 120 samples distributed in 40 patients (20 females and 20 males) with normal colonoscopy, 40 patients (20 females and 20 males) with advanced adenoma-AA (≥10 mm, villous histology, high-grade dysplasia) and 40 patients (20 females and 20 males) with CRC were selected. Cohort-study population characteristics are listed in the Supplementary Table S2.

### 4.2. Sample Preparation and Metabolomics Analysis

Frozen fresh fecal samples were shipped on dry ice to Metabolon, Inc. for UPLC-MS/MS analysis. Each sample was accessioned into the Metabolon Laboratory Information Management System (LIMS, Metabolon, Inc., Morrisville, NC, USA) and was assigned by the LIMS a unique identifier that was associated with the original source identifier only. This identifier was used to track all sample handling, tasks, results, etc. The samples (and all derived aliquots) were tracked by the LIMS system. All portions of any sample were automatically assigned their own unique identifiers by the LIMS [61] (see Supplementary Materials for more detailed information).

Samples were prepared using previous extraction methods by the automated MicroLab STAR^®^ system from Hamilton Company (MicroLab STAR^®^, Hamilton Robotics Inc., Reno, NV, USA) [76,77]. Then, proteins were removed by precipitating with methanol under vigorous shaking for 2 min in a GenoGrinder 2000 homogenizer (Glen Mills Inc., Clifton, NJ, USA) followed by centrifugation [76,77]. The resulting extract was divided into five aliquots, and the organic solvent was evaporated on a TurboVap^®^ (Zymark, Hopkinton, MA, USA) for analysis as it improves the chromatographic resolution, peak shape and compound detection [77]. The aliquots were used as follows: two for analysis by reverse-phase ultraperformance liquid chromatography–tandem mass spectrometry (UPLC-MS/MS) methods with positive-ion-mode electrospray ionization (ESI), one for analysis by reverse-phase UPLC-MS/MS with negative-ion-mode ESI, one for analysis by hydrophilic-interaction liquid chromatography (HILC) /UPLC-MS/MS with negative-ion-mode ESI, and one sample was reserved for backup [76,77,78] (see Supplementary Material for detailed information).

Metabolomic profiles were obtained by four different methods in a Waters ACQUITY ultraperformance liquid-chromatography system (UPLC) (Waters Corporation, Clifton, NS, USA) [77] coupled to a Q-Exactive high-resolution/accurate mass spectrometer (Thermo Scientific, Waltham, Mass, USA) [62] with heated electrospray ionization (HESI-II) source and operating at 35,000 mass resolution [75]. As different methods were used for the analysis, each dry extract was reconstituted in a compatible solvent to each method. Solvents contained a series of standards (isotopically labeled compounds) at fixed concentrations to monitor instrument performance, ensure data quality and serve as retention index markers for chromatographic alignment during data-processing step [77,79]. The UPLC and MS conditions are described in detailed by Ford L. et al. [78] (Supplementary Tables S1 and S2), and briefly explained in the Supplementary Material and Methods. The linearity associated with these methods was previously published and reported [80]. All the analyses previously mentioned were performed by Metabolon, Inc. 

In order to control and assess analytical variability during analysis, several quality samples were prepared (see Supplementary Material, QA/QC section, and Table S1). Instrument variability was determined by calculating the relative standard deviation (RSD) for the internal standards added to each sample prior to injection, and the overall process variability was determined by the RSD for all endogenous metabolites present in 100% of the pooled matrix sample. Both RSDs must fulfill the Metabolon acceptance criteria. Furthermore, in order to remove any time-related effects, samples were randomly injected in the sequence and the pooled matrix sample (QC) was also analyzed through the sequence. A scheme of the analytical sequence is included in Figure S1 (Supplementary Material).

### 4.3. Data Extraction and Compound Identification

Data extraction and compound identification were entirely performed by Metabolon, Inc. The information related to bioinformatics, LIMS, data extraction and compound identification is summarized in the Supplementary Material and is highly detailed in several published articles [78,80,81,82,83]. Briefly, raw data were extracted, peak-identified and QC processed using the Metabolon’s hardware and software (see Supplementary Material). Peak detection and integration were performed by the ThermoFisher Scientific (Waltham, MA, USA) software Xcalibur Quan Browser. Then, a list of *m/z* ratios, retention indices and areas under the curve (AUC) values were obtained. Afterwards, the biological data sets were chromatographically aligned based on the retention index that utilized internal standards assigned a fixed RI value. Finally, peaks were matched against the Metabolon´s in-house library of authentic standards, as well as compared with library entries of purified standards and routinely detected unknown compounds specific to the respective analytical method. 

The compound identification was based on three criteria: retention time index (window ≈ 10 s), experimental accurate mass match to the library authentic standards (±10 ppm), and the MS/MS forward and reverse scores between the experimental data and authentic standard. To each identified metabolite, an identification confidence level based on the MSI was assigned, being level 1 for those compounds validated with a pure standard; level 2 for compounds that were not confirmed by the standard but had been verified by MS/MS; level 3 for a tentative structure or a putative class; and level 4 for those unknown compounds (see Supplementary Table S1).

After compound identification, and before any statistical analysis, a curation step was also performed to remove background noise, artifacts, misassignments and to ensure accurate and consistent identification (see Supplementary Material).

### 4.4. Metabolite Quantification and Data Normalization

Once it was ensured that high-quality data had been obtained, peaks were quantified using AUC, and then data were normalized or corrected in run-day blocks by registering the medians equal to one and normalizing each data point proportionately (see Supplementary Material). Finally, missing values were imputed by the minimum value across all batches. 

### 4.5. Statistical Analysis for Metabolome and Clinical Data

R and JMP programs were used for the statistical analysis. Unsupervised analysis by principal component analysis (PCA, R function “prcomp”) was performed using the matrix that was previously median-scaled as well as log-transformed. This PCA model was used for data-dimension reduction, data visualization and group distribution or tendencies. This PCA was also utilized to understand global metabolic changes among control, advanced adenoma and CRC patients. Then, random forest (RF) (R package “randomforest”) [84] and logistic regression (R function “glm”) [85,86] were used to build supervised classification and prediction models. Considering the requirement of model validation bootstrapping with replacement was used, not only for supervised model validation, but also to minimize the bias and improve the precision of prediction [85] (see Supplementary Material for more details).

RF was further used to measure the importance of all variables and the ability of each variable to classify the data appropriately. In this sense, “Mean Decrease Accuracy” was used as the metric for variable importance selection. 

Finally, logistic regression, a generalized linear model of probability multivariate analysis that was used as a predictor of CRC, was used. From the database, the 120 samples were randomly sampled with replacements (bootstrapping method) splitting the dataset into two subsets by train-test split procedure to evaluate the performance model using the R package “caret” [87]. A training set with the size of 0.7 to build the prediction model and the remainder percentage 0.30 was assigned to the test set used as an evaluation model. Stepwise regression was built, in which all predictor variables were added or removed from the model one by one. Additionally, each step was tested to ensure the component’s significance [85,86]. This logistic regression was evaluated and validated with k-fold cross-validation of the generalized linear binomial model, completing left-one-out cross-validation (LOOCV) using the boot package. The area under receiver operating characteristic (ROC) curve (AUC) (R package “pROC” and “performance”) was used to evaluate the performance of the prediction model [88,89,90]. This procedure was repeated 1000 times, and the median of AUCs was regarded as the final AUC (R package “ROCR”) [89,90,91]. Analyses were performed using R software (version R 4.1.2) (Boston, MA, USA) [92].

Then, and considering the results obtained by PCA and RF, the univariate statistical test was applied. In this sense, Welch’s two-sample *t*-test was used to compare two-by-two groups and to identify metabolites that differed significantly between experimental groups [67]. Following the workflow detailed in the Supplementary Material, statistical significance was achieved as *q*-value ≤ 0.05, after applying multiple hypothesis-testing correction by the false-discovery rate (FDR). [76,86,93]. 

## Figures and Tables

**Table 1:** Significant metabolites obtained by Welch’s two-sample *t*-test followed by FDR (*q*-values ≤ 0.05), and fold-change heatmap, which indicates the ratio of the mean scaled intensity for each metabolite for the comparisons AA vs. Control (C), CRC vs. C, AA + CRC vs. C, CRC vs. AA, and C + AA vs. CRC. Red cells indicate that the mean values are significantly higher (upregulated) and green cells indicate the mean values are significantly lower (downregulated). MSI indicates the identification confidence level and C is the abbreviation of control group.

Pathway	Biochemical Name	AA vs. C		CRC vs. C		AA + CRC vs. C		CRC vs. AA		C + AA vs. CRC		MSI
		Fold Change	*q*-Value	Fold Change	*q*-Value	Fold Change	*q*-Value	Fold Change	*q*-Value	Fold Change	*q*-Value	
AMINO ACID												
Histidine Metabolism	formiminoglutamate	0.92	0.709	**2.02**	0.0817	1.47	0.5331	**2.21**	0.0089	**2.11**	0.0064	1
**PEPTIDE**												
Polypeptide	val-val-ala	0.51	0.731	**2.02**	0.0705	1.27	0.4626	**3.96**	0.0076	**2.68**	0.0064	1
	STVLT	0.46	0.8245	**11.83**	0.0065	**6.14**	0.241	**25.98**	0.0019	**16.26**	0.0022	1
**LIPID**												
Fatty Acid, Dicarboxylate	3-carboxy-4-methyl-5-propyl-2-furanpropanoate	1.3	0.7987	**2.78**	0.0339	**2.04**	0.2488	**2.13**	0.1472	**2.42**	0.0233	1
Fatty Acid Metabolism	eicosenoylcarnitine (C20:1)	0.73	0.6723	**0.41**	0.0063	**0.57**	0.241	0.57	0.7251	**0.48**	0.0274	2
Diacylglycerol	oleoyl-arachidonoyl-glycerol (18:1/20:4) [2] (DAG 38:5)	0.75	0.8245	**4.11**	0.0065	**2.43**	0.241	**5.5**	0.0017	**4.7**	0.0015	2
Ceramide	ceramide (d18:2/24:1, d18:1/24:2)	0.65	0.7016	**1.92**	0.0771	1.28	0.5331	**2.94**	0.0004	**2.32**	0.001	2
LacCer	lactosyl-N-palmitoyl-sphingosine (d18:1/16:0) (LacCer 34:1)	0.53	0.731	**3.06**	0.0631	1.8	0.4776	**5.78**	0.0013	**4,00**	0.0016	1
	lactosyl-N-nervonoyl-sphingosine (d18:1/24:1) (LacCer 42:3)	0.42	0.6788	**3.13**	0.1213	1.78	0.5463	**7.39**	0.0016	**4.4**	0.0041	2
Sphingomyelin (SM)	palmitoyl sphingomyelin (d18:1/16:0) (SM 34:1)	0.59	0.7225	**2.3**	0.0309	1.45	0.4556	**3.89**	0.001	**2.9**	0.001	1
	behenoyl sphingomyelin (d18:1/22:0) (SM 40:1)	0.56	0.6788	**2.04**	0.1068	1.3	0.5332	**3.64**	0.0021	**2.61**	0.005	2
	SM (d17:1/16:0, d18:1/15:0, d16:1/17:0)	0.52	0.6965	**1.89**	0.1643	1.2	0.5332	**3.67**	0.008	**2.49**	0.0233	2
	SM (d18:2/16:0, d18:1/16:1) (SM 34:2)	0.64	0.7359	**5.16**	0.0017	**2.9**	0.241	**8.01**	0.0001	**6.28**	0.0002	2
	SM (d18:1/20:0, d16:1/22:0) (SM 38:1)	0.45	0.7339	**1.63**	0.0779	1.04	0.461	**3.61**	0.0033	**2.25**	0.0064	2
	SM (d18:1/24:1, d18:2/24:0) (SM 42:2)	0.5	0.7186	**4.1**	0.0039	**2.3**	0.3232	**8.19**	0.00007	**5.46**	0.00008	2
	SM (d18:2/24:1, d18:1/24:2) (SM 42:3)	0.59	0.7359	**6.55**	0.0017	**3.57**	0.241	**11.19**	0.0001	**8.26**	0.0002	2
Secondary Bile Acid Metabolism	glycolithocholate sulfate	2.05	0.731	**0.28**	0.1213	1.17	0.5439	**0.14**	0.0332	**0.19**	0.0071	2
	glycocholenate sulfate	0.4	0.8598	**0.1**	0.1643	0.25	0.472	**0.24**	0.2052	**0.14**	0.0398	2
**NUCLEOTIDE**												
Pyrimidine Metabolism	cytidine	0.93	0.7359	**0.46**	0.0417	**0.7**	0.2488	**0.5**	0.3399	**0.48**	0.0398	1
**COFACTOR AND VITAMINS**												
Hemoglobin and Porphyrin Metabolism	heme	0.33	0.7604	**8.44**	0.0088	**4.38**	0.2885	**25.62**	0.0008	**12.69**	0.0011	1
	bilirubin (Z,Z)	0.52	0.7484	**0.16**	0.0813	**0.34**	0.3114	**0.31**	0.3557	**0.21**	0.0457	1
	bilirubin (E,E)	0.77	0.7849	**0.19**	0.1589	0.48	0.5331	**0.25**	0.0497	**0.21**	0.0105	2
**XENOBIOTICS**												
Xanthine Metabolism	3,7-dimethylurate	1.18	0.8245	**0.42**	0.125	0.8	0.461	**0.36**	0.1135	**0.39**	0.0398	1
**PARTIALLY CHARACTERIZED MOLECULES (PCM)**												
PCM	bilirubin degradation product, C16H18N2O5 (2)	0.91	0.7329	**0.31**	0.0219	**0.61**	0.2488	**0.34**	0.2451	**0.32**	0.0064	3
**UN NAMED**												
N/A	X-11787	1.28	0.8318	**3.57**	0.0065	**2.43**	0.241	**2.78**	0.0127	**3.13**	0.0027	4

## Data Availability

The data presented in this study are available on request from the corresponding author. The data are not publicly available due to the methodology is under intellectual properties issues.

## Supplementary Materials

The following supporting information can be downloaded at: https://www.mdpi.com/article/10.3390/metabo12060550/s1. Table S1: Description of Metabolon quality control samples; Table S2: Metabolon quality control standards; Figure S1. Preparation of technical replicates; Figure S2: Visualization of data normalization steps for a multiday platform run.; Figure S3. Ranfom Forest Confusion Matrix obtained by bootstrapping for the three groups (Control (C), AA and CRC); Figure S4. Random Forest Confusion Matrix obtained by bootstrapping for the fusion of AA + CRC vs. Control (A), and for the fusion of AA + Control vs. CRC (B)
