# Supplementary material for: A Comprehensive Metabolomics Analysis of Fecal Samples from Advanced Adenoma and Colorectal Cancer Patients
Source: Metabolites. 2022 Jun 15;12(6):550. doi: 10.3390/metabo12060550 (PMC9229737; doi:10.3390/metabo12060550)
Supplement: Supplementary file 1 [file metabolites-12-00550-s001.zip › Supplementary Material.pdf]

Supplementary Material for:

A comprehensive metabolomics analysis of fecal samples from advanced adenoma and colorectal cancer patients

Authors: Oiana Telleria[1]*, Oihane E. Alboniga[2], Marc Clos-Garcia[3], Beatriz Nafría-Jimenez[4], Joaquin Cubiella[5], Luis Bujanda[6] and Juan Manuel Falcón-Pérez[1,2,7]*

Methodology (performed by Metabolon, Inc.)

Sample Accessioning: Following receipt, samples were inventoried and immediately stored at -80 ºC. Each sample received was accessioned into the Metabolon Laboratory Information System (LIMS, Metabolon, Inc., Morrisville, NC, US) and a unique identifier, which was associated with the original source only, was assigned. This identifier was used to track all sample handling, tasks, results, etc. The samples (and all derived aliquots) were tracked by the LIMS system. All portions of any sample were automatically assigned their own unique identifiers by the LIMS when a new task was created; the relationship of these samples was also tracked. All samples were maintained at -80 ºC until processed and subsequent analysis.

Sample Preparation: Frozen fecal samples were prepared using the automated MicroLab STAR® system from Hamilton Company. Several recovery standards were added prior to the first step in the extraction process for quality control purposes. Then, protein precipitation was performed by adding methanol. After vigorous shaking for 2 min, samples were centrifuged, and supernatant was divided in 5 aliquots. Since samples were analyzed by four different methods each aliquot was used as follows: two for the analysis by reverse phase ultraperformance liquid chromatography-tandem mass spectrometry (UPLC-MS/MS) methods with positive ion mode electrospray ionization (ESI), one for analysis by reverse-phase UPLC-MS/MS with negative ion mode ESI, one for analysis by hydrophilic interaction liquid chromatography (HILC) /UPLC-MS/MS with negative ion mode ESI, and one sample was reserved for backup. The aliquots were placed briefly on a TurboVap® (Zymark) to remove the organic solvent. Sample extracts were stored overnight under nitrogen before preparation for analysis. Finally, on analysis day, dry extracts were reconstituted in a compatible solvent to each method. Solvents contained a series of standards (isotopically compounds) at fixed concentrations to monitor instrument performance, ensure data quality and serve for chromatographic alignment during data processing.

QA/QC: Several types of controls were analyzed in concert with the experimental samples: a pooled matrix sample generated by taking a small volume of each experimental sample that served as a technical replicate throughout the data set (CMTRX) from now called QC sample; extracted water samples served as process blanks (PRCS); and a cocktail of QC standards, that were carefully chosen not to interfere with the measurement of endogenous compounds, were spiked into every analyzed sample (QA),and allowed instrument performance monitoring as well as aided chromatographic alignment. Tables S1 and S2 describe these samples and standards. Instrument variability was determined by calculating the median relative standard deviation (RSD) for the standards that were added to each sample prior to injection into the mass spectrometers. Overall process variability was determined by calculating the median RSD for all endogenous metabolites (i.e., non-instrument standards) present in 100 % of the QCs pooled matrix samples. Experimental samples were randomized across the platform run with QC samples spaced evenly among the injections, as outlined in Figure S1.

Table S1: Description of Metabolon quality control samples

Type	Description	Purpose
MTRX	Large pool of human plasma maintained by Metabolon that has been characterized extensively.	Assure that all aspects of the Metabolon process are operating within specifications.
CMTRX	Pool created by taking a small aliquot from every customer sample.	Assess the effect of a non-plasma matrix on the Metabolon process and distinguish biological variability from process variability.
PRCS	Aliquot of ultra-pure water	Process Blank used to assess the contribution to compound signals from the process.
SOLV	Aliquot of solvents used in extraction.	Solvent Blank used to segregate contamination sources in the extraction.

Table S2: Metabolon quality control standards

Type	Description	Purpose
RS	Recovery Standard	Assess variability and verify performance of extraction and instrumentation.
IS	Internal Standard	Assess variability and performance of instrument.

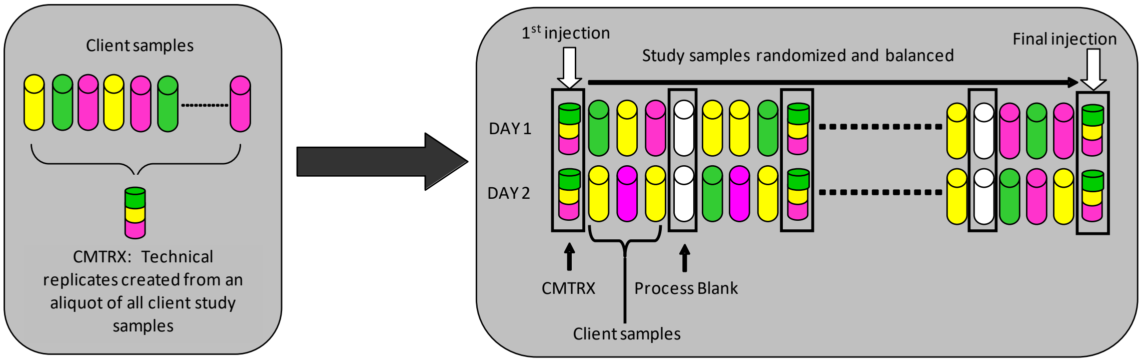

Figure S1. Preparation of technical replicates. A small aliquot of each sample (colored cylinders) is pooled to create a CMTRX (QC) technical replicate sample (multi-colored cylinder), which is then injected periodically throughout the platform run. Variability among consistently detected biochemicals can be used to calculate an estimate of overall process and platform variability.

Ultrahigh Performance Liquid Chromatography-Tandem Mass Spectroscopy (UPLC-MS/MS) analysis: All methods utilized a Waters ACQUITY ultra-performance liquid chromatography (UPLC) coupled to a Thermo Scientific Q-Exactive high resolution/accurate mass spectrometer interfaced with a heated electrospray ionization (HESI-II) source and operating at 35,000 mass resolution. Considering the reconstituted aliquots in different solvents previously mentioned, each aliquot was analyzed as follows. One aliquot was analyzed using acidic positive ion conditions, chromatographically optimized for more hydrophilic compounds. In this method, the extract was gradient eluted from a C18 column (Waters UPLC BEH C18-2.1x100 mm, 1.7 µm) using water and methanol, containing 0.05 % perfluoropentanoic acid (PFPA) and 0.1 % formic acid (FA). Another aliquot was also analyzed using acidic positive ion conditions, however it was chromatographically optimized for more hydrophobic compounds. In this method, the extract was gradient eluted from the same afore mentioned C18 column using methanol, acetonitrile, water, 0.05 % PFPA and 0.1 % FA and was operated at an overall higher organic content. Another aliquot was analyzed using basic negative ion optimized conditions using a separate dedicated C18 column. The basic extracts were gradient eluted from the column using methanol and water, however with 6.5 mM ammonium bicarbonate at pH 8. The fourth aliquot was analyzed via negative ionization following elution from a HILIC column (Waters UPLC BEH Amide 2.1x150 mm, 1.7 µm) using a gradient consisting of water and acetonitrile with 10 mM ammonium formate, pH 10.8. The MS analysis alternated between full scan MS and data-dependent MS^n scans using dynamic exclusion. The scan range varied slighted between methods but covered 70-1000 m/z. Raw data files are archived and extracted as described below.

Bioinformatics: The informatics system consisted of four major components, the Laboratory Information Management System (LIMS), the data extraction and peak-identification software, data processing tools for quality control and compound identification, and a collection of information interpretation and visualization tools for use by data analysts. The hardware and software foundations for these informatics components were the LAN backbone, and a database server running Oracle 10.2.0.1 Enterprise Edition.

LIMS: The purpose of the Metabolon LIMS system was to enable fully auditable laboratory automation through a secure, easy to use, and highly specialized system. The scope of the Metabolon LIMS system encompasses sample accessioning, sample preparation and instrumental analysis,reporting and advanced data analysis. All of the subsequent software systems are grounded in the LIMS data structures. It has been modified to leverage and interface with the in-house information extraction and data visualization systems, as well as third party instrumentation and data analysis software.

Data Extraction and Compound Identification: Raw data were extracted, peak-identified and QC processed using Metabolon's hardware and software. These systems are built on a web-service platform utilizing Microsoft's .NET technologies, which run on high-performance application servers and fiber-channel storage arrays in clusters to provide active failover and load-balancing. Compounds were identified by comparison to library entries of purified standards or recurrent unknown entities. Metabolon maintains a library based on authenticated standards that contains the retention time/index (RI), mass to charge ratio (*m/z)*, and chromatographic data (including MS/MS spectral data) on all molecules present in the library. Furthermore, biochemical identifications are based on three criteria: retention index within a narrow RI window of the proposed identification (≈ 10 s), accurate mass match to the library +/- 10 ppm, and the MS/MS forward and reverse scores between the experimental data and authentic standards. The MS/MS scores are based on a comparison of the ions present in the experimental spectrum to the ions present in the library spectrum. While there may be similarities between these molecules based on one of these factors, the use of all three data points can be utilized to distinguish and differentiate biochemicals. More than 3300 commercially available purified standard compounds have been acquired and registered into LIMS for analysis on all platforms for determination of their analytical characteristics.

Additional mass spectral entries have been created for structurally unnamed biochemicals, which have been identified by virtue of their recurrent nature (both chromatographic and mass spectral). These compounds have the potential to be identified by future acquisition of a matching purified standard or by classical structural analysis.

Curation: A variety of curation procedures were carried out to ensure that a high-quality data set was made available for statistical analysis and data interpretation. The QC and curation processes were designed to ensure accurate and consistent identification of true chemical entities, and to remove those representing system artifacts, mis-assignments, and background noise. Metabolon data analysts use proprietary visualization and interpretation software to confirm the consistency of peak identification among the various samples. Library matches for each compound were checked for each sample and corrected if necessary.

Metabolite Quantification and Data Normalization: Peaks were quantified using area-under-the-curve (AUC). As the study requires multiple days, a data normalization step was performed to correct variation resulting from instrument inter-day tuning differences. Essentially, each compound was corrected in run-day blocks by registering the medians to equal one (1.00) and normalizing each data point proportionately (termed the "block correction"; Figure S2).

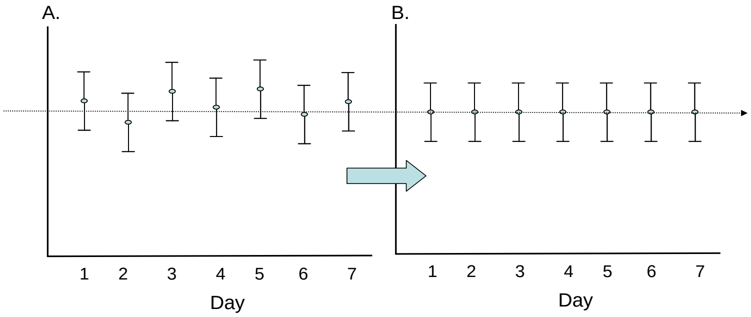

Figure S2: Visualization of data normalization steps for a multiday platform run.

Statistical Methods and Terminology

Statistical Calculations: Two types of statistical analysis were performed: (1) significance tests (Welch's two-sample t-test) and (2) classification analysis. Standard statistical analyses are performed in the programs R (http://cran.r-project.org/) and JMP.

Firstly, principal component analysis (PCA), an unsupervised analysis, was used to reduce data dimension and improved data visualization. PCA was also used for the assessment of analytical quality, system reproducibility, and group tendency. Afterwards, for variable selection, a supervised method was employed. Among them, Random Forest (based on decision trees) was selected for classification as it does not overfit compared to other supervised methods and it is invariant to transformation. Data were split on two sets in order to create or train the model and to test the model. The first one consisted of a training set used to train the model and generate classifications (70 % of samples) and the second one "out-of-bag" (OOB) or test set was used for class prediction (remaining 30 % of samples). This process was performed by bootstrapping with replacement, and it was repeated 1000 times. Finally, OOB error rate was used as a measure of prediction accuracy as a confusion matrix. This confusion matrix and the prediction accuracy obtained is an unbiased estimation of how well the classification model can predict sample class in a new data set.

To determine which variables (metabolites) make the largest contribution to the classification, a "variable importance" measure is computed. We use the "Mean Decrease Accuracy" (MDA) as this metric. The MDA is determined by randomly permuting a variable, running the observed values through the trees, and then reassessing the prediction accuracy. If a variable is not important, then this procedure will have little change in the accuracy of the class prediction (permuting random noise will give random noise). By contrast, if a variable is important to the classification, the prediction accuracy will drop after such a permutation, which we record as the MDA. Thus, the RF analysis provides an "importance" rank ordering of metabolites; we typically output the top 30 compounds in the list as potentially worthy of further investigation.

Finally, logistic regression, which is a non-linear regression, was used as a predictor of CRC patients. This logistic regression was evaluated and validated by boot package using k-fold cross validation of generalized linear binomial model completing leave one out cross validation (LOOCV). The performance of the prediction was evaluated by the area under receiver operating characteristic (ROC) curve (AUC).

Once all metabolites were studied at the same time by multivariate analysis, one by one were studied by univariate statistical analysis. Considering that the study includes three groups, ANOVA should be used, but as it can be seen in the manuscripts, two by two comparisons were performed and thus Welch' two-sample t-test was used. Whelch's t-test (*p*-value ≤0.05) was used to establish whether the metabolite was significant when two groups were studied. In all cases, false positive rate was corrected by the false discovery rate (FDR) at a level of α = 0.05 (*q*-value ≤0.05) to reduce the number of false significant metabolites. In this case, it is considered a significant metabolite when *q*-value was less than 0.05.

Random Forest Confusion Matrix

Predicted Groups

Actual Groups

Feces	C	AA	CRC	Class Error
C	19	18	3	0.525
AA	15	20	5	0.500
CRC	6	10	24	0.400
Predictive accuracy = 52%				

Note that random segregation would give a predictive accuracy of 33%.

Figure S3. Ranfom Forest Confusion Matrix obtained by bootstrapping for the three groups (Control (C), AA and CRC).

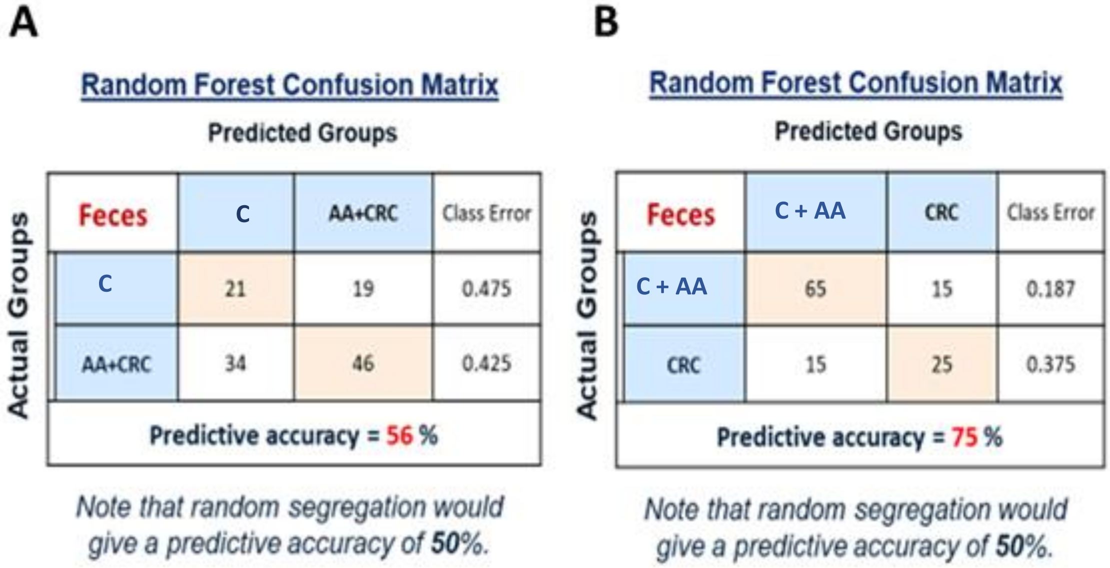

Figure S4. Random Forest Confusion Matrix obtained by bootstrapping for the fusion of AA + CRC vs Control (A), and for the fusion of AA + Control vs CRC (B).
